# Didang Tang alleviates neuronal ferroptosis after intracerebral hemorrhage by modulating the PERK/eIF2α/ATF4/CHOP/GPX4 signaling pathway

**DOI:** 10.3389/fphar.2024.1472813

**Published:** 2024-10-24

**Authors:** Jing Lu, Hanying Xu, Li Li, Xiaolei Tang, Ying Zhang, Dongmei Zhang, Peng Xu, Liwei Sun, Jian Wang

**Affiliations:** ^1^ Research Center of Traditional Chinese Medicine, The Affiliated Hospital to Changchun University of Chinese Medicine, Jilin, China; ^2^ College of Traditional Chinese Medicine, Changchun University of Chinese Medicine, Jilin, China; ^3^ Department of Encephalopathy, The Affiliated Hospital of Changchun University of Chinese Medicine, Jilin, China; ^4^ Nursing Department, The Affiliated Hospital to Changchun University of Chinese Medicine, Jilin, China; ^5^ Scientific Research Office, The Affiliated Hospital to Changchun University of Chinese Medicine, Jilin, China

**Keywords:** Didang Tang, ferroptosis, intracerebral hemorrhage, endoplasmic reticulum stress, HPLC

## Abstract

**Introduction:**

Ferroptosis is a crucial process contributing to neuronal damage following intracerebral hemorrhage (ICH). Didang Tang (DDT), a traditional therapeutic, has been used clinically to manage ICH for many years, yet the molecular mechanisms by which by DDT protects neurons from ferroptosis after ICH remain elusive.

**Methods:**

This study utilized high-performance liquid chromatography-based fingerprint analysis to characterize DDT’s chemical composition. An ICH rat model and hemin and erastin-induced PC12 cell ferroptosis models were developed to investigate DDT’s neuroprotective mechanisms. Histological assessments of brain tissue morphology and iron deposition were performed using hematoxylin-eosin, Nissl, and Perl’s blue staining. Neurological function was evaluated using Longa and Berderson scores, while lipid peroxidation was measured using biochemical assays and flow cytometry. Protein expression levels of ferroptosis- and endoplasmic reticulum stress (ERS)-related markers were analyzed via Western blotting and immunofluorescence.

**Results:**

Our results demonstrated that DDT reduced hematoma volume, decreased iron deposition, lowered malondialdehyde (MDA) levels, and upregulated glutathione peroxidase (GPX4) and SLC7A11 expression in affected brain regions. Furthermore, DDT downregulated GRP78 expression and inhibited the PERK/eIF2α/ATF4/CHOP/GPX4 pathway, exerting strong neuroprotective effects. The fluorescence staining results of MAP2/GPX4 and MAP2/CHOP suggested that DDT may regulate neuronal ferroptosis and ERs to exert the protective effect. *In vitro* experiments using hemin- and erastin-induced neuron-derived PC12 cells as neuronal ferroptosis models developed in our laboratory corroborated these *in vivo* findings, showing increased survival and reduced lipid peroxidation in DDT-treated cells, along with similar inhibitory effects on ferroptosis and ERS. Molecular docking suggested that DDT components, such as sennoside B, amygdalin, rhein, and emodin, interact favorably with PERK/eIF2α/ATF4/CHOP signaling pathway proteins, highlighting their potential role in DDT’s anti-ferroptosis effects.

**Conclusion:**

DDT alleviates neuronal ferroptosis after ICH by modulating the PERK/eIF2α/ATF4/CHOP/GPX4 signaling pathway. Overall, this study provides novel insights into DDT’s protective mechanisms against ICH-induced neuronal injury by modulating ferroptosis and ERS pathways, underscoring its potential as an effective therapeutic strategy.

## 1 Introduction

Intracerebral hemorrhage (ICH) accounts for approximately 15%–20% of all strokes, and the majority of survivors suffer from lifelong neurological deficits (Li et al., 2022; Liddle et al., 2023). P Primary brain damage results from hematomas, followed by secondary damage arising from blood degradation products, inflammation, and edema (Butcher et al., 2004). Surgical hematoma evacuation remains the primary intervention for ICH but provides limited therapeutic benefits while posing risks of secondary damage (De Oliveira Manoel, 2020). Despite pharmacotherapeutic advancements, current drug treatments remain insufficient in achieving satisfactory outcomes (Gross et al., 2019). Although the precise mechanisms of ICH-induced brain injury are not fully understood, dysregulated iron metabolism is thought to play a pivotal role in the pathogenesis of this disorder (Sun et al., 2022).

Ferroptosis, a unique form of programmed cell death first identified by Dixon’s research group in 2012, is characterized by enhanced iron-dependent lipid peroxidation (Dixon et al., 2012). It is defined by disrupted glutathione (GSH) antioxidant system function, depletion of glutathione peroxidase 4 (GPX4), and the subsequent accumulation of lipid peroxides (Hu et al., 2019). During ICH, a significant release of iron ions into brain tissue occurs within 24 h and persists for up to 28 days, primarily due to the breakdown of hemoglobin into iron, biliverdin, and carbon monoxide by heme oxygenase (HO-1) (Zille et al., 2017) with excess iron contributing to peripheral edema and peroxide accumulation (Chen et al., 2019). When lipid accumulation exceeds the antioxidant capacity of cells, cytotoxic compounds are generated, leading to protein denaturation, lipid damage, and neuronal death. Emerging evidence suggests that ferroptosis inhibitors may reduce secondary brain damage following ICH (Li et al., 2017), highlighting the potential of ferroptosis-targeted therapies in mitigating ICH-induced brain injury.

Drawing from traditional Chinese medical theory, the *Treatise on Blood Syndromes* (“Xue Zheng Lun”) describes hemorrhagic stroke as a form of blood stasis (“After bleeding, even if the blood is fresh and clear, it is still considered stagnant blood”), with the obstruction of cerebral collaterals considered its fundamental cause. Clinically, this theory has been validated, indicating that blood stasis persists throughout the course of ICH. Consequently, treatments that promote blood circulation and eliminate blood stasis are thought to facilitate the absorption of stagnant blood, thereby clearing cerebral collaterals and restoring normal cerebral circulation.

DiDang Tang (DDT), originating from the *Treatise on Cold Pathogenic Diseases*, is composed of four traditional Chinese medicines: peach seed (*Prunus persica* (L.) Batsch), rhubarb (*Rheum palmatum* L.), gadflies (*Tabanus bivittatus* Matsumura), and leeches (*Whitmania pigra* Whitman). Each component has distinct therapeutic properties: leeches effectively break down pathological blood stasis without damaging new blood; gadflies and peach seed promote blood circulation and resolve stasis, while rhubarb dispels stasis and clears heat. The combination of these ingredients forms a potent formula for resolving blood stasis and improving circulation. Pharmacologically, DDT has demonstrated antioxidant, anti-apoptotic, and neuroprotective properties across various disease models (Huang et al., 2018; Lu et al., 2020). Recent studies suggest that DDT effectively ameliorates hemorrhagic brain injury (Lu et al., 2022), with some of its active components shown to reduce iron deposition (Kuo et al., 2020; Lin et al., 2021). Given these findings, we hypothesized that DDT might alleviate neural dysfunction in ICH by inhibiting ferroptosis. Therefore, this study aims to investigate how DDT exerts neuroprotective effects against ferroptosis in ICH-induced brain injury.

## 2 Materials and methods

### 2.1 Preparation of DiDang Tang (DDT) extract

**FIGURE 1:**
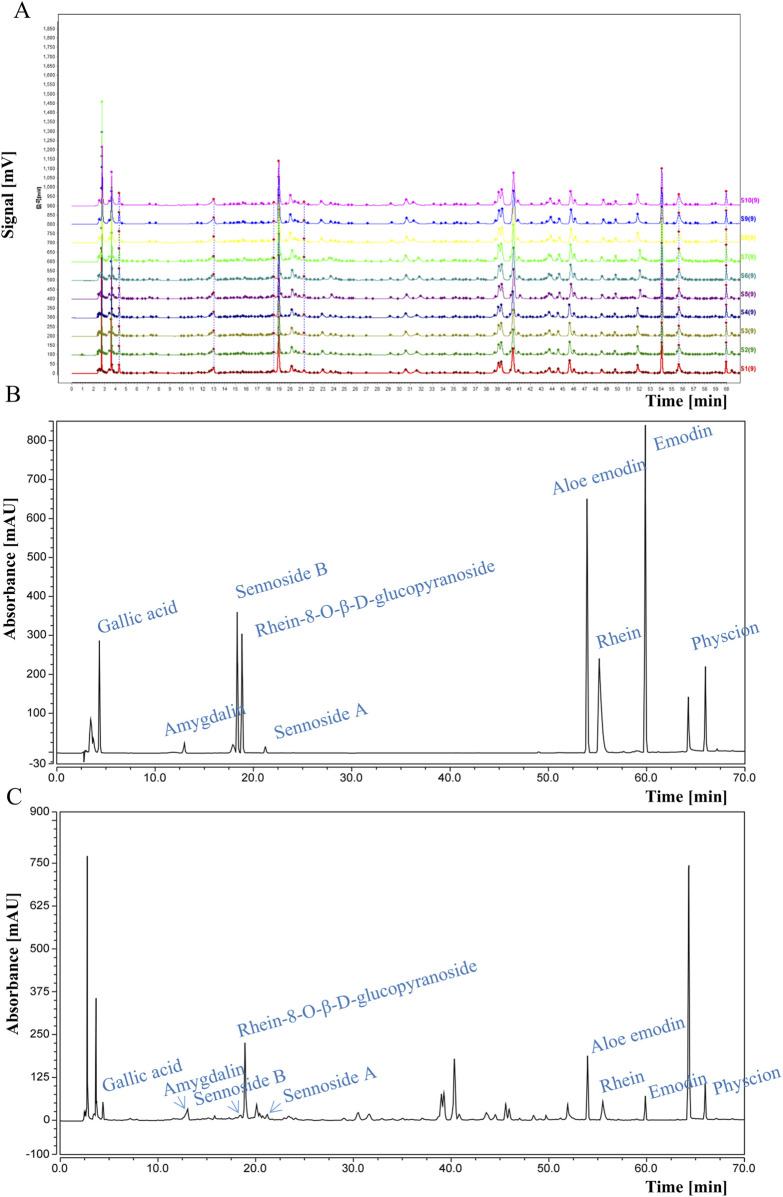
HPLC analysis of DDT. **(A)** HPLC fingerprints of 10 batches of DDT. **(B)** HPLC chromatograms of mixed standards. **(C)** HPLC chromatographic profile of DDT.

DDT samples were procured from the Affiliated Hospital of Changchun University of Traditional Chinese Medicine. The formula comprises four components: rhubarb, leeches, peach seeds, and gadflies in a weight ratio of 5:3:10:3. Briefly, the mixture was soaked for 30 min in water at 10 times its volume, followed by three additional extractions at 100°C for 30 min each, which were pooled and centrifuged. The supernatant was retained, filtered, and freeze-dried, generating a brown-colored powder with a yield of 33.1%. Quality control of the extract was performed via high-performance liquid chromatography (HPLC) using a flow rate of 0.9 mL/min, with the column temperature maintained at 25°C, and detection performed at 254 nm. HPLC fingerprint analysis was conducted using the Chinese Medicine Chromatographic Fingerprint Similarity Evaluation System (2012 Edition), showing a similarity index of >0.98 across all 10 batches, demonstrating that our DDT extraction method is reproducible (Figure 1A). Retention time analysis was further validated using reference standards of amygdalin, rhein, rhein-8-O-β-D-glucopyranoside, gallic acid, sennoside B, aloe emodin, sennoside A, physcion, and emodin (Figure 1B). Nine characteristic peaks corresponding to these standards were observed (Figure 1C).

### 2.2 Animal studies

Male Sprague-Dawley rats (250–300 g) were obtained from the Experimental Animal Center of Jilin University, Changchun, China. All experimental procedures were approved by the Experimental Animals Committee of Changchun University of Chinese Medicine (approval no. 2020131), and conducted following the European Community Guidelines/EEC Directive of 1986. Seventy-five rats were randomly assigned to five groups (n = 15 per group): Sham, ICH, and three DDT treatment groups with doses per body weight of 0.13 g/100 g, 0.26 g/100 g, and 0.52 g/100 g, administered orally 2 h post-ICH induction, once daily for 7 consecutive days.

The ICH models were established as previously described (Kong et al., 2021). Rats were anesthetized with isoflurane and immobilized using a stereotaxic frame for ICH or sham surgery, and their body temperature was maintained at 37°C during surgery. To collect autologous blood, gently warm the rat’s tail using a heat lamp for 3 min to dilate the veins. Disinfect the tail with 70% ethanol. Using a sterile 25-gauge needle, puncture the tail vein and draw approximately 50 µL of blood into a sterile syringe. After blood collection, carefully remove the needle and apply gentle pressure to the puncture site with sterile gauze to stop any bleeding. The collected blood should be retained in the syringe to prevent coagulation. A burr hole was drilled 3 mm lateral to the right of the midline and 0.4 mm posterior to the bregma, through which 50 µL of autologous blood was slowly infused into the brain using a 26-gauge needle inserted at a depth of 6.5 mm below the skull surface. Infusion was conducted over 5 min to minimize tissue trauma of surrounding brain tissue, thereby mimicking the natural course of ICH. The burr hole was sealed with bone wax, and the wound was sutured. After the rats woke up, they were provided free access to water and food. During the experiment, 2 rats in the ICH group and 1 rat each in the 0.26 g/100 g and 0.52 g/100 g DDT groups died.

### 2.3 Hematoxylin-eosin (HE) staining

Brain tissues were paraffin-embedded, sectioned, and dewaxed using standard protocols (Liu X. et al., 2021). Slices were immersed in hematoxylin for 3 min at 25°C, rinsed, and destained using an alcohol/hydrochloric acid mixture for 30 s, followed by thorough rinsing in a stream of water until the blue color returned. Tissues were next treated with 95% ethanol, stained with acidified eosin ethanol, and dehydrated with ethanol and xylene. Finally, the slices were sealed with neutral balsam, then observed structure and morphology of brain tissues around the hematoma using an optical microscope (Canon SX20).

### 2.4 Evaluation of longa neurological deficits

Neurological deficits were assessed using the Longa neurological deficit score (Liu C. D. et al., 2021). Scoring criteria were defined as 0, no deficits; 1, inability to fully extend the contralateral forelimb; 2, contralateral circling; 3, falling to the contralateral side while walking; 4, reduced consciousness and inability to walk. Rats scoring 0 or 1 during the initial evaluation were excluded. Two independent, blinded evaluators performed the scoring.

### 2.5 Berderson score

The rat was lifted 10 cm off the surface by its tail. Normal rats extend both forelimbs, while those with neurological deficits show various degrees of deformity (Song et al., 2022). A three-point scoring system was used: 0, no nerve damage; 1, flexion of wrist and elbow joints and adduction of the shoulder; 2, decreased thrust on the paralyzed side; 3, circling behavior towards the affected side. Each test was repeated three times per rat.

### 2.6 Nissl staining

Neuronal morphology around the hematoma was observed using Nissl staining. After processing the paraffin sections, they were stained for 10 min using Nissl solution and rinsed twice with distilled water. The number of Nissl-positive neurons was quantified in five randomly selected fields per section using an optical microscope (Shi et al., 2021).

### 2.7 Perl’s blue staining

Perl’s blue staining was performed to assess iron deposition in the hematoma and surrounding areas (Wang et al., 2022). Sections were treated with Perl’s blue staining solution for 20 min, dehydrated with ethanol, destained until transparent, and sealed according to the manufacturer’s protocol.

### 2.8 Biochemical analysis

Brain tissues around the hematoma were lysed in RIPA buffer, and the levels of superoxide dismutase (SOD), glutathione peroxidase (GSH-px), GSH, the reduced glutathione (GSH) to oxidized glutathione (GSSG) ratio (GSH/GSSH), and MDA were determined using assay kits as per the manufacturer’s instructions (Mughal et al., 2017).

### 2.9 Western blotting

Cells and brain tissues were lysed using RIPA buffer. Proteins (40 µg) were separated using 10% or 12% SDS-PAGE and transferred to PVDF membranes. After blocking with 5% non-fat milk, membranes were incubated overnight with primary antibodies at 4°C, followed by secondary antibody incubation for 1 h. Protein bands were visualized using a chemiluminescence imaging system (Tang et al., 2021).

### 2.10 PC12 cell culture

PC12 cells were cultured in RPMI 1640 medium containing 5% FBS, 10% horse serum, and 1% penicillin-streptomycin at 37°C with 5% CO₂. For neurite induction, cells were treated with 50 ng/mL nerve growth factor for 24 h prior to experiments (Lu et al., 2020).

### 2.11 Cell viability assay

Following treatment with DDT or hemin/erastin for 24 h, 10 μL of cell counting kit-8 (CCK8) reagent was added to each well, and the absorbance was measured at 450 nm (Tan et al., 2019).

### 2.12 Flow cytometry analysis of Fe^2+^ and lipid peroxidation in PC12 cells

Cells treated with hemin/erastin and/or varying concentrations of DDT for 24 h were incubated with 5 μM FeRhoNox-1 for 60 min at 37°C in the dark, after which Fe^2^⁺ levels were analyzed using flow cytometry. Lipid peroxidation was then assessed by adding C11 BODIPY 581/591 followed by a 30-minute incubation at 37°C (Homma et al., 2019).

### 2.13 Immunofluorescence staining

Brain tissue sections and fixed cell coverslips were blocked with 5% normal donkey serum and permeabilized with 0.3% Triton X-100. The sections were then incubated with primary antibodies (1:200) overnight at 4°C. After incubation with fluorescent secondary antibodies (1:1,000) for 1 h, cells were counterstained with 4′,6-diamidino-2-phenylindole (DAPI) for 10 min. The fluorescent staining in the brain tissue surrounding the hematoma and the cell coverslips were observed using an immunofluorescence microscope (Gamdzyk et al., 2020). Each experimental group was represented by three samples.

### 2.14 Molecular docking

Libdock scores showed binding ability between core ingredients and hub targets, were calculated using Discovery Studio 2019 (DS 2019). Chemical structures of key DDT components (amygdalin, rhein, rhein-8-O-β-D-glucopyranoside, gallic acid, sennoside B, aloe emodin, sennoside A, physcion, and emodin) used as ligands in molecular docking analysis were downloaded from the PubChem database (https://pubchem.ncbi.nlm.nih.gov/). The 3D structures of four target proteins (PERK, EIF2A, ATF4, CHOP) were retrieved from the RCSB PDB database (https://www.pdb.org/). Rigid protein–protein docking (ZDOCK) was performed between Hirudin and hub targets to study the relationships.

### 2.15 Statistical analysis

Data from three independent experiments were analyzed using GraphPad Prism 6.0 and expressed as the mean ± standard deviation. One-way ANOVA followed by Tukey’s *post hoc* test was used for comparisons, with a *p*-value <0.05 considered statistically significant.

## 3 Results

### 3.1 DDT reduced nerve injury in rats with ICH

**FIGURE 2:**
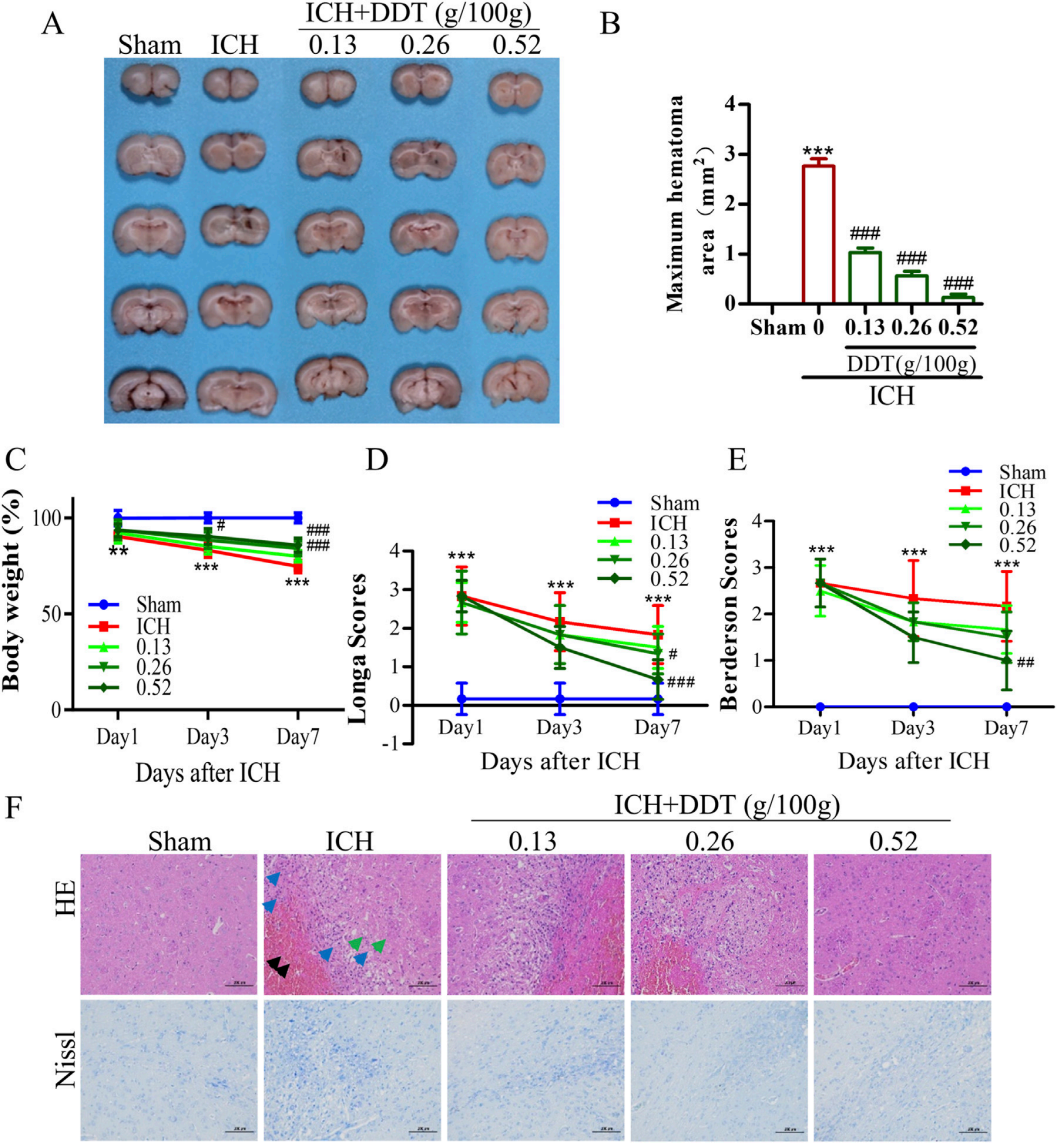
DDT reduced nerve injury in rats with ICH. **(A)** Representative images of hemorrhagic lesions in rats from different groups. **(B)** The quantitative analysis of hematoma volume. **(C)** Changes in body weight across the groups. **(D, E)** Longa and Berderson scores of ICH rats with/without DDT treatment at 1, 3 and 7 days post-ICH. **(F)** Representative images showing HE and Nissl staining of brain sections across the groups. Scale bar = 100 μm ^**^ *p* < 0.01, ^***^ *p* < 0.001 compared to the Sham group; ^#^ *p* < 0.05, ^##^ *p* < 0.01, ^###^ *p* < 0.001 compared to the ICH model group.

To investigate the neuroprotective effects of DDT following ICH, we established an ICH rat model and treated it with DDT. As shown in Figures 2A, B, the hematoma was significantly reduced in the model group, while DDT treatment reduced the hematoma in a dose-dependent manner. The body weight of rats significantly decreased after ICH, but it markedly increased after treatment with DDT starting on the third day post-injury, with the most pronounced effect observed on day 7 (Figure 2C). ICH rats exhibited severe neurological dysfunction, with significantly increased Longa and Berderson scores. After treatment with DDT, these scores decreased markedly on day 7, indicating a gradual improvement in neurological function (Figures 2D, E). In subsequent studies, we conducted additional assessments on day 7, when the effects of DDT were most pronounced. HE staining further revealed that the brain tissue of the Sham group had a dense population of morphologically normal-appearing neurons that were uniformly distributed, with no obvious glial cell proliferation. In contrast, the ICH group showed localized blood clots in the striatum, composed of erythrocytes and insoluble fibrin (black arrow), along with numerous macrophages and lymphocytes (blue arrows), disorganized neurons, and tiny round vacuoles (green arrows). Following DDT treatment, the presence of erythrocytes and inflammatory infiltration was significantly reduced, and neurons were more orderly arranged with increasing DDT doses (Figure 2F). Nissl staining corroborated these observations: the Sham group displayed normal neurons, evenly arranged, while the ICH group exhibited disrupted neuronal morphology, nerve substance coagulation, and irregularly degenerated neurons around the hematoma. DDT treatment markedly improved neuronal morphology and organization (Figure 2F).

### 3.2 DDT reduced ferroptosis around hematoma after ICH

**FIGURE 3:**
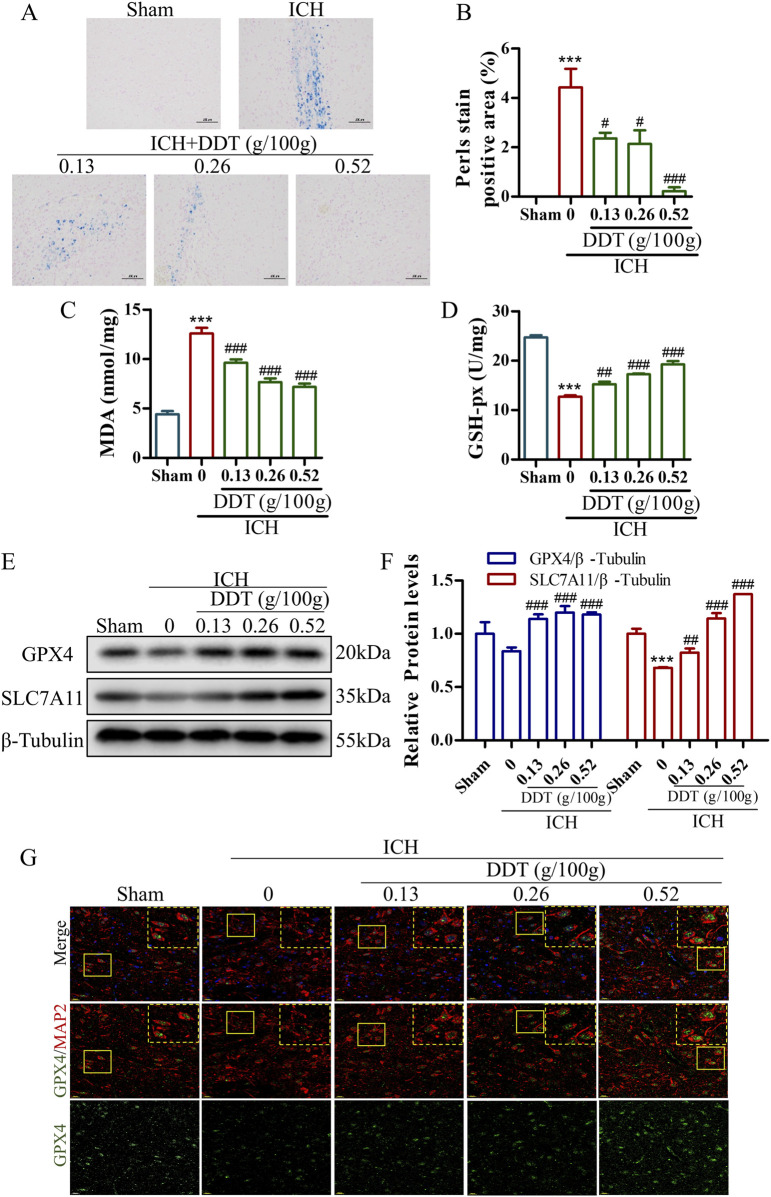
DDT reduced ferroptosis around hematoma after ICH. **(A)** Representative images of brain sections stained with Prussian blue from different experimental groups. Scale bar = 100 μm. **(B)** Quantitative analysis of Prussian blue-positive areas across the different groups. **(C, D)** MDA and GSH-px levels in brain tissue surrounding hematomas of different groups. **(E)** Protein levels of GPX4 and SLC7A11 around the hematoma at 7 days post-ICH. **(F)** Quantifications of GPX4 and SLC7A11 expression levels in rats. **(G)** Immunofluorescence staining of MAP2/GPX4 expression in brain tissue surrounding hematomas of different groups. Scale bar = 20 μm ^***^ *p* < 0.001 compared to the Sham group; ^#^ *p* < 0.05, ^##^ *p* < 0.01, ^###^ *p* < 0.001 compared to the ICH model group.

Given that ferroptosis is closely associated with iron accumulation and typically induced by lipid peroxidation, the levels of iron and MDA were estimated in brain tissues post-ICH. Representative images of Perl’s blue-stained tissues indicated significant iron deposition around hematomas in ICH rats, which was nearly eliminated following treatment with the administration of 0.5 g/100 g DDT (Figures 3A, B). Concurrently, MDA levels, which were significantly elevated in ICH rats, were markedly reduced in perihematomal tissues following DDT treatment (Figure 3C). At the same time, GSH-px levels, which were profoundly decreased in the ICH group, were significantly increased with DDT treatment (Figure 3D). Moreover, Western blot analysis showed a significant reduction in the expression of key ferroptosis markers, GPX4 and solute carrier family 7 member 11 (SLC7A11), in the ICH group. This downregulation was effectively reversed by DDT administration (Figures 3E, F). To further localize DDT effects, we performed immunofluorescence analysis using microtubule-associated protein 2 (MAP2) to label neurons and observed GPX4 expression in neuronal cells. DDT treatment significantly restored the expression of GPX4 in neuronal cells (Figure 3G). Collectively, these findings demonstrate that DDT attenuates neuronal ferroptosis in the perihematomal region by reducing iron accumulation and lipid peroxidation, thereby exerting a protective effect after ICH.

### 3.3 DDT alleviated endoplasmic reticulum stress (ERS) in perihematomal tissues in ICH rats

**FIGURE 4:**
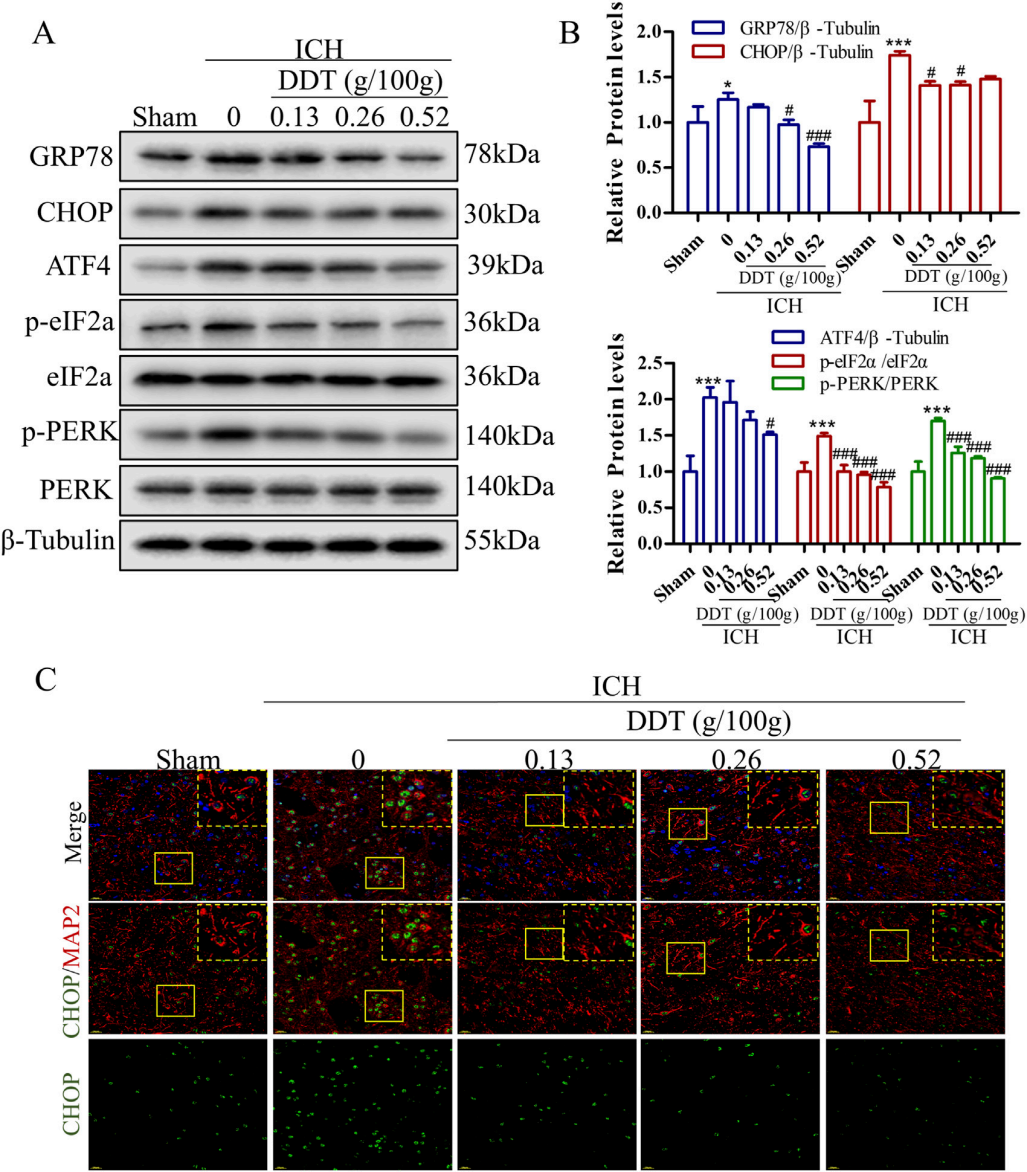
DDT alleviated ERS around hematomas after ICH. **(A)** Protein levels of GRP78, CHOP, ATF4, p-PERK, PERK, p-eIF2α, and eIF2α, around hematomas of different groups at 7 days post-ICH. **(B)** Quantitative analysis of protein expression levels in **(A)**. **(C)** Immunofluorescence staining of MAP2/CHOP in brain tissue surrounding hematomas of different groups. Scale bar = 20 μm ^*^ *p* < 0.05, ^***^ *p* < 0.001 compared to the Sham group; ^#^ *p* < 0.05, ^###^ *p* < 0.001 compared to the ICH model group.

Research has shown that ERS plays a pivotal role in the progression of ferroptosis. Our findings revealed that the expression of ERS markers GRP78 and CHOP was significantly increased in the ICH group but was markedly decreased following DDT treatment (Figures 4A, B). Moreover, the expression levels of phosphorylated protein kinase R (PKR)-like endoplasmic reticulum kinase (p-PERK), phosphorylated eukaryotic initiation factor 2 alpha (p-eIF2α), and activating transcription factor 4 (ATF4) were significantly elevated in the ICH group but downregulated significantly after DDT administration (Figures 4A, B). Further immunofluorescence analysis indicated that CHOP expression levels were specifically diminished in neurons (MAP2^+^ cells) following ICH. The results demonstrated that DDT specifically inhibited CHOP expression in neurons (Figure 4C). In summary, these findings suggest that DDT alleviates ERS in the perihematomal region, particularly in neurons, by regulating the PERK/eIF2α/ATF4 signaling pathway.

### 3.4 DDT inhibited ferroptosis in hemin-induced PC12 cells

**FIGURE 5:**
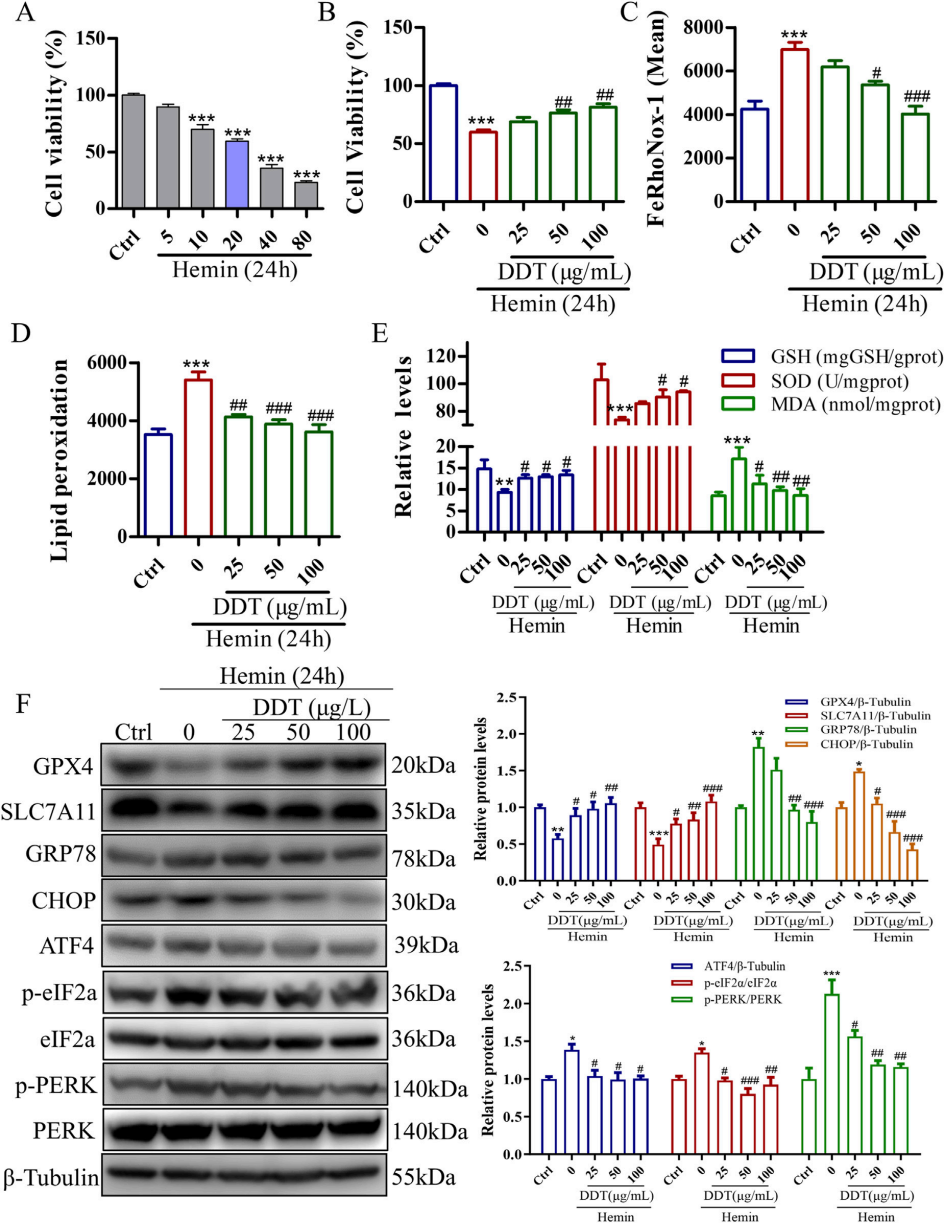
DDT inhibited ferroptosis in PC12 cells induced by exposure to hemin. **(A)** The effect of hemin induction on cell viability in PC12 cells, evaluated using the CCK8 assay. **(B)** PC12 cells were treated with 20 μM hemin for 24 h, followed by DDT treatment for an additional 24 h. Cell viability was then assessed using the CCK8 assay. **(C)** Intracellular Fe^2^⁺ accumulation in hemin-induced PC12 cells was measured using the FeRhoNox-1 fluorescent probe, and the mean value of Fe^2^⁺ accumulation is shown. **(D)** Lipid peroxidation levels were evaluated using a C11 BODIPY probe and analyzed by flow cytometry (FCM). The bar chart shows the mean values of lipid peroxidation levels. **(E)** GSH-px, SOD, and MDA levels in PC12 cells. **(F)** Protein levels of GPX4, SLC7A11, GRP78, CHOP, ATF4, p-PERK, PERK, p-eIF2α, eIF2α analyzed by Western blotting. Quantitative analysis is shown on the right. ^*^ *p* < 0.05, ^**^ *p* < 0.01, ^***^ *p* < 0.001 compared to the Ctrl group; ^#^ *p* < 0.05, ^##^ *p* < 0.01, ^###^ *p* < 0.001 compared to the hemin group.

To further validate DDT neuroprotective effects observed in the animal experiments, we established a hemin-induced PC12 cell model to mimic the neuronal damage caused by ICH. As shown in Supplementary Figure S1A, no cytotoxic effects were observed in PC12 cells treated with DDT at concentrations below 200 μg/mL for 24 h. We further assessed Fe^2^⁺ accumulation to determine the appropriate dosing concentration, as it is the most direct indicator of ferroptosis. The results indicated that the most pronounced effects of DDT were observed at concentrations of 25, 50, and 100 μg/mL (Supplementary Figure S1B). Therefore, we selected these three concentrations for subsequent experiments. Using a CCK8 assay, we found that 20–80 μM of hemin significantly decreased cell viability (Figure 5A), which was significantly restored following DDT treatment (Figure 5B). Flow cytometry revealed that DDT significantly reduced hemin-induced Fe^2^⁺ accumulation (Figure 5C) and lipid peroxidation levels (Figure 5D). Moreover, DDT markedly decreased the content of MDA, a lipid peroxidation marker, while significantly enhancing the activities of antioxidant enzymes GSH-px and SOD (Figure 5E). Western blot analysis further demonstrated that DDT treatment significantly upregulated the expression of ferroptosis-inhibitory proteins GPX4 and SLC7A11 (Figure 5F), consistent with *in vivo* findings. Furthermore, DDT notably suppressed the expression of key ERS proteins GRP78, CHOP, ATF4, p-PERK, and p-eIF2α (Figure 5F). These results indicate that DDT effectively inhibits hemin-induced ferroptosis in PC12 cells, potentially by modulating the PERK/eIF2α/ATF4 pathway.

### 3.5 Erastin-induced ERS and ferroptosis in PC12 cells

**FIGURE 6:**
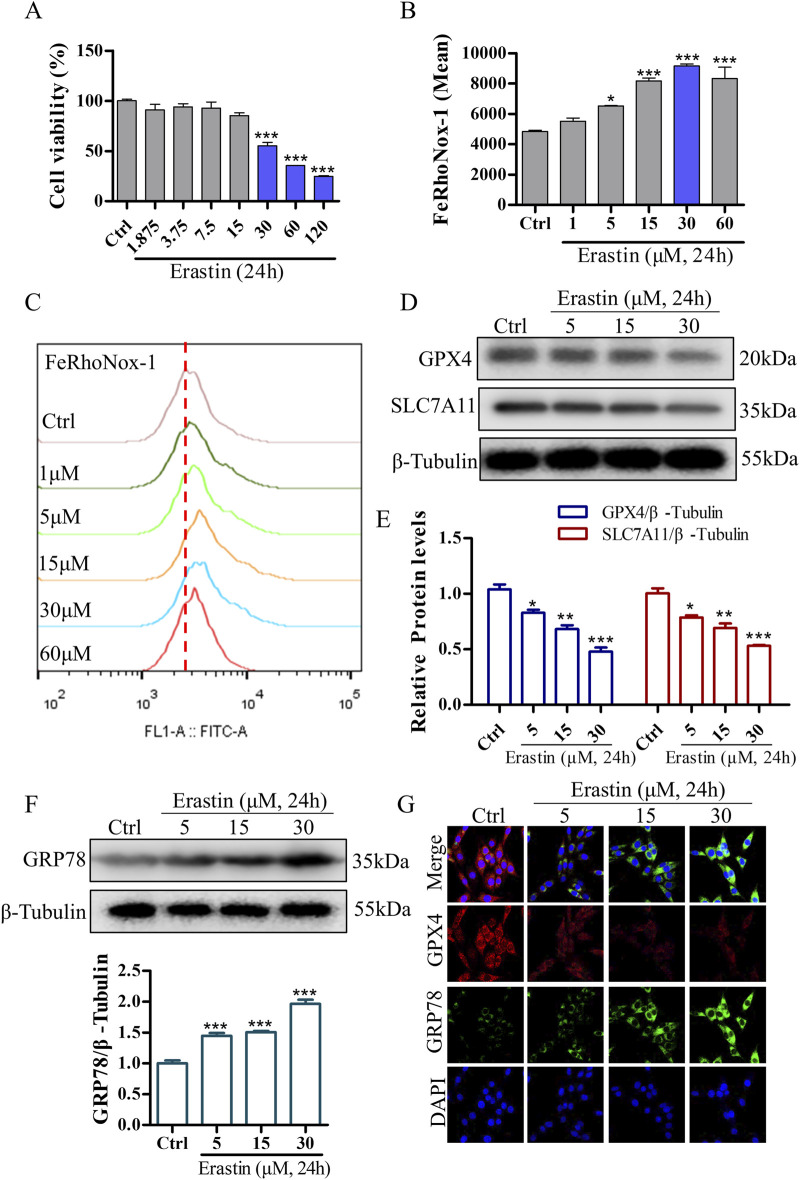
Erastin induces ERS and ferroptosis in NGF-differentiated PC12 cells. **(A)** The effect of erastin-induced cytotoxicity on cell viability in PC12 cells as measured using the CCK8 assay. **(B, C)** Erastin-induced Fe^2+^ accumulation in PC12 cells was evaluated using the FeRhoNox-1 fluorescent probe, followed by FCM. Mean values of Fe^2+^ accumulation were shown. **(D)** Protein levels of GPX4 and SLC7A11 in PC12 cells treated with varying concentrations of erastin, analyzed using Western blotting. **(E)** Quantitative analysis of protein expression in **(D)**. **(F)** GRP78 expression in PC12 cells treated with different concentrations of erastin, analyzed using Western blotting, with relative levels shown below. **(G)** Immunofluorescence staining of GRP78 and GPX4 in PC12 cells following erastin treatment. ^*^ *p* < 0.05, ^**^ *p* < 0.005, ^***^ *p* < 0.001 compared to the Ctrl group.

To validate the neuroprotective effects of DDT in inhibiting ferroptosis, we used erastin to establish a ferroptosis model in PC12 cells. Treatment with erastin led to a significant reduction in cell viability (Figure 6A) and induced a dose-dependent increase in intracellular Fe^2+^ accumulation (Figures 6B, C). To optimize the erastin concentration for inducing ferroptosis, we evaluated the expression levels of key ferroptosis-associated proteins, including GPX4 and SLC7A11. The results demonstrated a significant, dose-dependent decrease in GPX4 and SLC7A11 expression compared with the Ctrl group (Figures 6D, E, G). Additionally, we examined whether erastin-induced ferroptosis is mediated through ERS by measuring levels of GRP78, an ER chaperone that dissociates from transmembrane receptors to initiate the unfolded protein response (UPR). As shown in Figures 6F, G, GRP78 levels were significantly elevated in response to erastin concentrations ranging from 5 to 30 μM, especially at 30 μM. These findings suggest that erastin induces ferroptosis through ERS in PC12 cells. Based on these results, PC12 cells were incubated with 30 μM erastin for 24 h as the optimal protocol for inducing the cellular ferroptosis model in subsequent experiments.

### 3.6 DDT inhibited ferroptosis in erastin-induced PC12 cells

**FIGURE 7:**
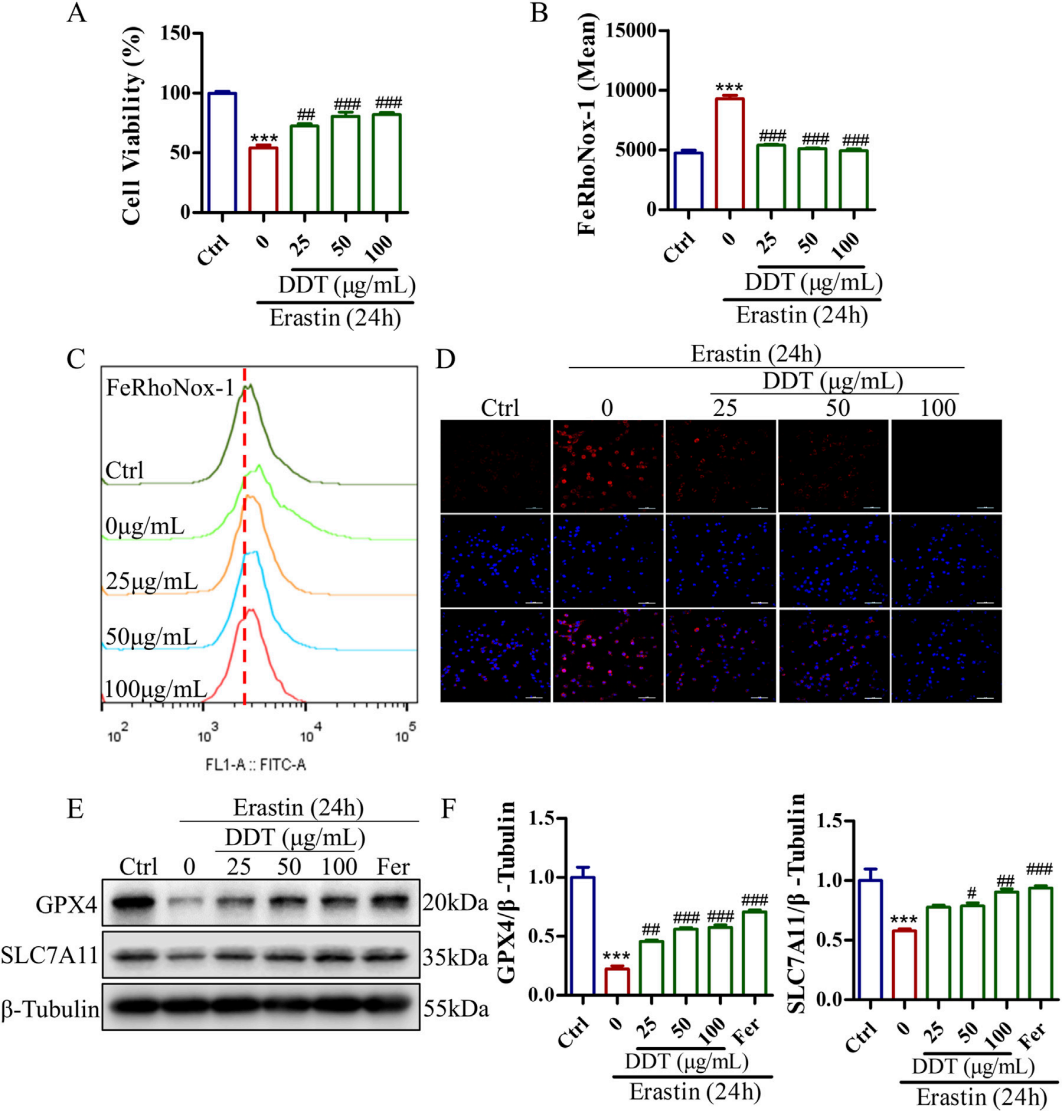
DDT inhibited ferroptosis in PC12 cells following erastin exposure. **(A)** PC12 cells were treated with DDT followed by 30 μM erastin for 24 h, after which cell viability was assessed using the CCK8 assay. **(B, C)** Cells were stained with FeRhoNox-1 fluorescent probe and analyzed by FCM. The bar chart shows the mean values of intracellular Fe^2+^ accumulation. **(D)** Immunofluorescence staining of Fe^2+^ in PC12 cells. **(E)** Protein levels of GPX4 and SLC7A11 in PC12 cells treated with erastin and/or varying concentrations of DDT, analyzed by Western blotting. **(F)** Quantitative analysis of protein levels in **(E)**. ^***^ *p* < 0.001 compared to the Ctrl group; ^#^ *p* < 0.05, ^##^ *p* < 0.01, ^###^ *p* < 0.001 compared to the erastin group.

To further investigate the protective effects of DDT against erastin-induced ferroptosis *in vitro*, we assessed cell viability, Fe^2+^ accumulation, and the expression levels of key ferroptosis-related proteins. As shown in Supplementary Figure S1C, the most pronounced effects of DDT were observed at concentrations of 25, 50, and 100 μg/mL, which were selected for subsequent experiments. As depicted in Figure 7A, erastin treatment significantly reduced cell viability to 54.14%. DDT administration restored cell viability in a dose-dependent manner to 72.40%, 80.54%, and 82.05% for low, medium, and high DDT doses, respectively. Flow cytometry and immunofluorescence analyses showed that DDT significantly alleviated erastin-induced Fe^2+^ accumulation (Figures 7B–D). Western blot analysis further revealed that erastin significantly downregulated GPX4 and SLC7A11 levels compared to the Ctrl group, while DDT treatment significantly upregulated their expression compared to the model group (Figures 7E, F). As a positive control, ferrostatin-1 (Fer), a known ferroptosis inhibitor, produced regulatory effects similar to those observed in the high-dose DDT group. Collectively, these findings demonstrate that DDT effectively inhibits erastin-induced ferroptosis in PC12 cells, potentially by restoring GPX4 and SLC7A11 expression and mitigating Fe^2^⁺ accumulation.

### 3.7 DDT inhibited lipid peroxidation in erastin-induced PC12 cells

**FIGURE 8:**
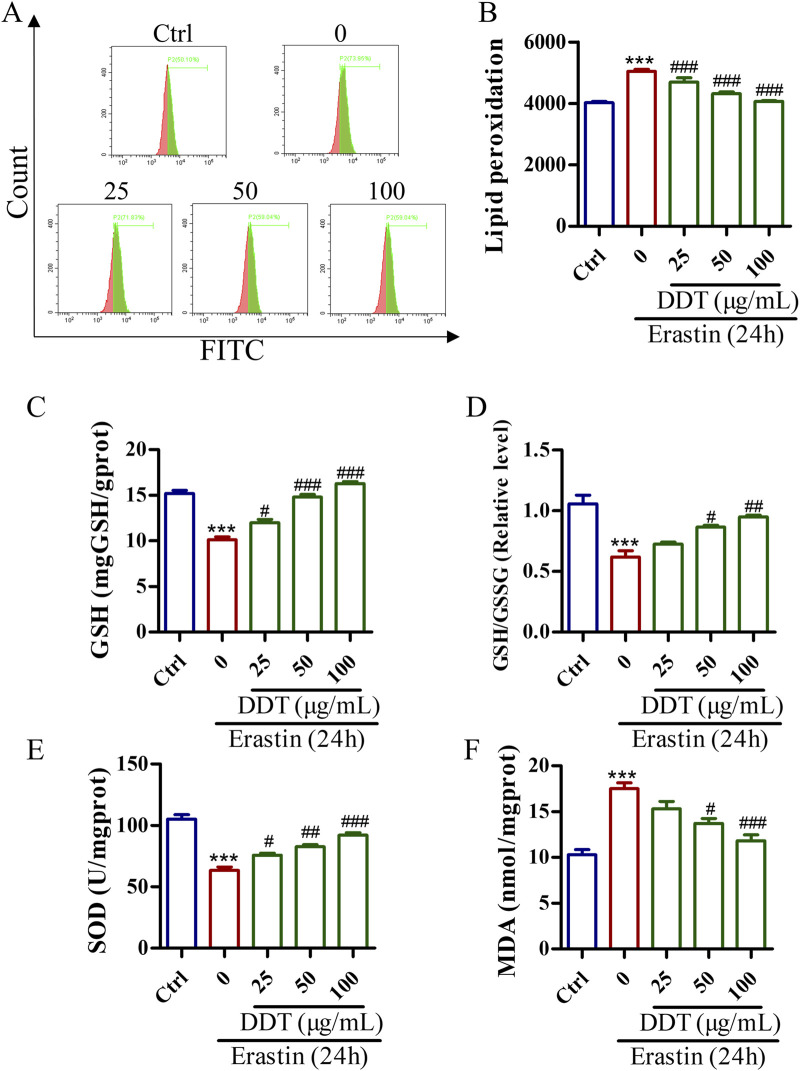
DDT inhibited lipid peroxidation in PC12 cells undergoing erastin-induced ferroptosis. **(A)** Cells were incubated with a C11 BODIPY probe to assess lipid peroxidation using FCM. **(B)** Bar chart displaying mean values of lipid peroxidation level in PC12 cells. **(C)** GSH levels, **(D)** GSH/GSSG ratios, **(E)** SOD levels. **(F)** MDA levels in PC12 cells undergoing ferroptosis. ^***^ *p* < 0.001 compared to the Ctrl group; ^#^ *p* < 0.05, ^##^ *p* < 0.01, ^###^ *p* < 0.001 compared to the erastin group.

Lipid peroxidation is a crucial initiator of ferroptosis. The evaluate the protective effects of DDT against erastin-induced lipid peroxidation, a series of experiments was conducted. Flow cytometry analysis showed that DDT treatment significantly suppressed erastin-induced lipid peroxidation (Figures 8A, B). Additionally, as shown in Figure 8C, erastin treatment decreased the GSH content from 15.19 to 10.11 mg/g protein, while DDT treatment dose-dependently restored GSH content to 11.99, 14.80, and 16.27 mg/g protein, respectively. Furthermore, erastin treatment visibly reduced the GSH/GSSG ratio and SOD content compared to the Ctrl group, while DDT treatment significantly elevated GSH/GSSG and SOD levels compared to the erastin-induced group (Figures 8D, E). In parallel, erastin induction markedly elevated MDA levels in PC12 cells, which were significantly mitigated by DDT treatment in a dose-dependent manner (Figure 8F). Collectively, these results indicate that DDT effectively reduced lipid peroxidation and restored the redox balance in erastin-induced PC12 cells.

### 3.8 DDT decreased erastin-induced ERS by blocking PERK signaling in PC12 cells

**FIGURE 9:**
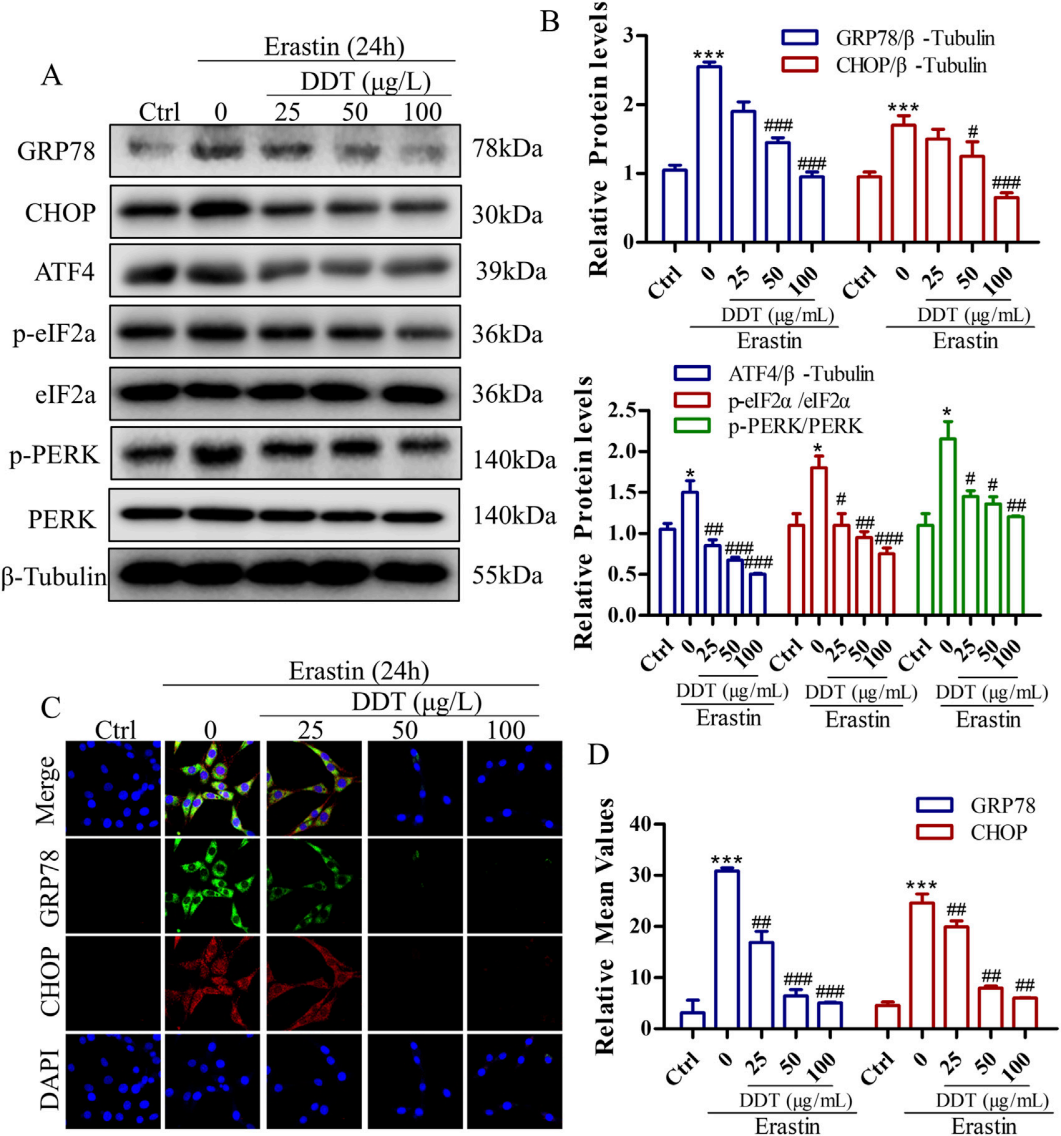
DDT Decreased erastin-induced ER Stress Through Blocking PERK Signaling in PC12 Cells. **(A)** Protein levels of GRP78, CHOP, ATF4, p-eIF2α, eIF2α, p-PERK, and PERK were observed by Western blotting. **(B)** Quantitative analysis of protein expression in **(A)**. **(C)** Immunofluorescence staining of GRP78 and CHOP after DDT treatment in PC12 cells. **(D)** Mean fluorescence intensity values are shown. ^*^ *p* < 0.05, ^***^ *p* < 0.001 compared to the Ctrl group; ^#^ *p* < 0.05, ^##^ *p* < 0.01, ^###^ *p* < 0.001 compared to the erastin group.

To further elucidate the mechanism by which DDT modulates ERS in the context of ferroptosis, we examined the expression of key ERS-related proteins. As depicted in Figures 9A–D, erastin treatment led to significantly increased the levels of GRP78 and CHOP proteins compared to the Ctrl group, indicating robust ERS induction by erastin. Notably, DDT treatment significantly reduced the expression levels of GRP78, CHOP, ATF4, p-PERK, and p-eIF2α compared to the Ctrl group. These findings suggest that DDT markedly mitigated ERS induced by erastin in PC12 cells by inhibiting the PERK/eIF2α/ATF4 pathway, thereby attenuating ERS-related damage.

### 3.9 Molecular docking analysis

**FIGURE 10:**
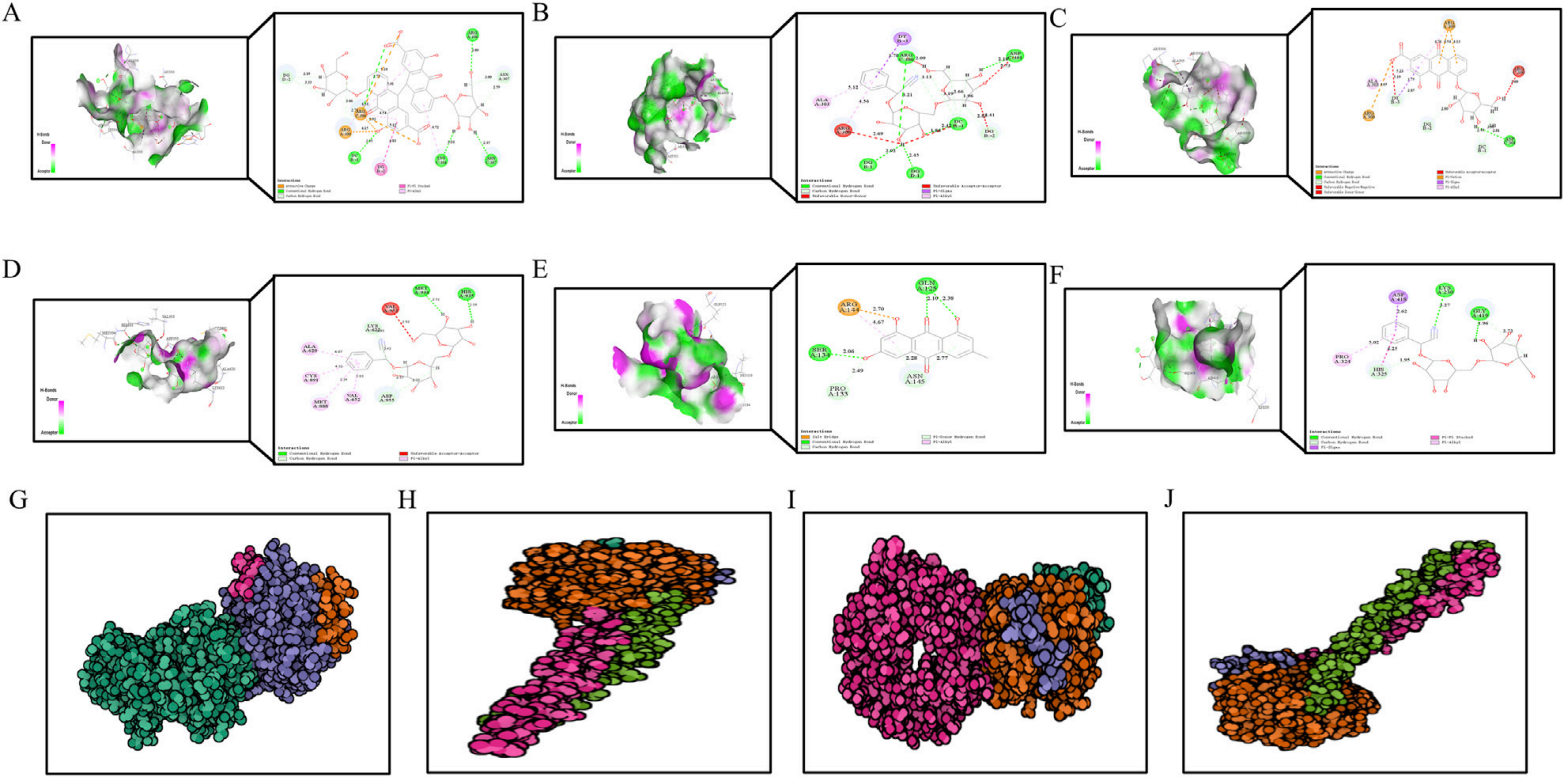
The diagram of molecular docking analysis. The diagram of molecular docking analysis. **(A–F)** 3D and 2D docking patterns of hub targets and key compounds: **(A)** Sennoside B-CHOP (Libdock:171.729). **(B)** Amygdalin-CHOP (Libdock:158.539). **(C)** Rhein-8-O-β-D-Glucopyranodise-CHOP (Libdock:133.385). **(D)** Sennoside B-PERK (Libdock:131.101). **(E)** EIF2A-Emodin (Libdock:126.429). **(F)** EIF2A-Sennoside B (Libdock:119.174). Lines of different colors represent various interactions. (Orange lines represent attractive charge or salt bridge interactions, Dark pink lines indicate pi-pi stacked interactions, Green lines show conventional hydrogen bonds, Light pink lines represent pi-alkyl interactions, Light green lines correspond to carbon or pi-donor hydrogen bonds, Purple lines signify pi-sigma interactions, Red lines denote unfavorable interactions, such as acceptor-acceptor, donor-donor, or negative-negative interactions.) The higher the Libdock score, the better the binding energy of hub genes and key compounds of herbs. **(G–I)** 3D docking patterns of hub targets and Hirudin (PDB ID: 16131390) the active protein in leech: **(G)** Hirudin-PERK (PDB ID: 4X7K, ZDock-Score:1279.557). **(H)** Hirudin-ATF4 (PDB ID: 8DYS, ZDock-Score:1279.984). **(I)** Hirudin-EIF2A (PDB ID: 1CI6, ZDock-Score:1286.565) **(J)** Hirudin-CHOP (PDB ID: 1NMQ, ZDock-Score:1098.362). The higher the ZDock score the better the binding energy of hub genes and Hirudin. Different atoms are identified as different colors, green is carbon, red is oxygen, and blue is nitrogen.

To further investigate the interaction between DDT components and ERS-related targets, molecular docking analysis was conducted. Crystal structures of the target proteins—including PERK, EIF2α, ATF4, and CHOP—were downloaded from the RCSB PDB database. DS 2019 software used to assess the stability and validity of the docking configurations. Docking was performed between these receptors and key DDT bioactive compounds. As shown in Figures 10A–F, Libdock scores were utilized to quantify binding efficiencies, with higher scores indicating more potent and stable protein-ligand interactions. The nine main bioactive compounds exhibited exemplary binding affinities with the key targets. The six most notable binding efficiencies were as follows: Sennoside B with CHOP (Libdock Score = 171.729), Amygdalin with CHOP (Libdock Score = 158.539), Rhein-8-O-β-D-glucopyranoside with CHOP (Libdock Score = 133.385), Sennoside B with PERK (Libdock Score = 131.101), EIF2A with Emodin (Libdock Score = 126.429), and EIF2A with Sennoside B (Libdock Score = 119.174). Hirudin, the active protein in leeches and a key component of DDT, was evaluated using ZDock scores to assess the binding energy with hub targets; higher ZDock scores indicate better binding energy. The results presented in Figures 10G–J demonstrate stable binding interactions among Hirudin and the proteins PERK, ATF4, EIF2A, and CHOP, with corresponding ZDock Scores of 1279.557, 1279.984, 1286.565, and 1098.362, respectively. The above results support the potential mechanism by which DDT regulates the PERK/eIF2α/ATF4 pathway to inhibit ferroptosis.

## 4 Discussion

In this study, we explored the neuroprotective effects of DDT in mitigating ferroptosis, a type of programmed cell death, following ICH. Our results demonstrated that DDT significantly reduced hematoma volume and improved neurological function *in vivo*. Additionally, both *in vivo* and *in vitro* experiments confirmed that DDT effectively suppressed neuronal ferroptosis by inhibiting iron deposition and lipid peroxidation, the primary drivers of ferroptosis, through upregulation of GPX4 and SLC7A11, which reduce lipid peroxides and maintain antioxidant defenses, respectively. Furthermore, DDT alleviated ERS, a critical contributor to ferroptosis development. Notably, our findings showed that DDT’s effect in reducing ferroptosis was dependent on its upregulation of GPX4 and SLC7A11 expression through the modulation of the PERK/eIF2α/ATF4 pathway. Molecular docking results further indicated that specific bioactive ingredients within DDT, such as sennoside B, amygdalin, rhein, and emodin, exhibited strong binding affinities to key regulatory proteins such as PERK, eIF2α, ATF4, and CHOP. These interactions suggest that DDT may inhibit the activation of the PERK/eIF2α/ATF4/CHOP signaling cascade by directly targeting these proteins, further supporting its role in ferroptosis inhibition. These findings suggest that DDT holds significant therapeutic potential for treating ferroptosis-related neuronal damage post-ICH.

Ferroptosis, a recently identified form of programmed cell death, has been increasingly recognized as a critical process contributing to neuronal damage following ICH due to iron-dependent lipid peroxidation (Li et al., 2009). Our results align with previous studies identifying ferroptosis as a key contributor to secondary brain injury following ICH (Zhang et al., 2022), and demonstrating that excessive iron deposition and lipid peroxidation exacerbate neuronal damage in ICH (Li et al., 2021). Moreover, ferroptosis inhibitors have been shown to effectively reduce ferroptosis-related damage (Li et al., 2017). This study, however, provides new evidence demonstrating the potential of a traditional Chinese medicine formula, DDT, in regulating the PERK/eIF2α/ATF4/CHOP pathway to inhibit ferroptosis. Our molecular docking studies suggest that DDT directly targets multiple key proteins in this pathway. Specifically, docking analyses revealed strong binding between DDT ingredients and the kinase domain of PERK, potentially inhibiting its activation and subsequent phosphorylation of eIF2α. Furthermore, DDT’s interactions with ATF4 and CHOP may reduce their transcriptional activity, thereby mitigating ERS-induced ferroptosis. In addition, our findings extend our current understanding of GPX4’s critical role in ferroptosis regulation. Previous studies have highlighted the importance of GPX4 in inhibiting ferroptosis by reducing lipid peroxides, which aligns with our findings showing that DDT upregulates GPX4 and SLC7A11 expression (Liu et al., 2018). Importantly, DDT’s inhibitory effects on ERS-related proteins, such as GRP78 and CHOP, distinguish it from conventional ferroptosis inhibitors that primarily target oxidative stress and iron metabolism. These findings offer new insights into the molecular mechanisms by which DDT combats ferroptosis, suggesting its potential as a promising therapeutic approach for managing ICH-induced neuronal injury.

The protective effects of DDT against ICH-induced neuronal ferroptosis can be attributed to its regulation of multiple signaling pathways, particularly the PERK/eIF2α/ATF4/CHOP/GPX4 axis. ERS is a critical factor in the initiation of ferroptosis, driven by the accumulation of unfolded proteins and iron overload, leading to neuronal death through the activation of the UPR pathway (Cominacini et al., 2015; Mohammed Thangameeran et al., 2020). Our docking experiments demonstrated that DDT components should effectively bind to the active site of PERK, inhibiting its activation. In turn, this inhibition likely prevents the phosphorylation of eIF2α, a key step in activating ATF4 (Rozpedek et al., 2016), while DDT’s binding to CHOP reduces its ability to induce downstream apoptosis and ferroptosis.

The combination of these molecular interactions supports DDT’s potent inhibitory effects on ferroptosis by attenuating both ERS and oxidative stress. In this study, DDT significantly downregulated the expression of GRP78, p-PERK, p-eIF2α, ATF4, and CHOP, thereby mitigating ERS and inhibiting ferroptosis. By preventing the activation of CHOP, DDT likely preserves GPX4 activity, a key enzyme that protects neurons from oxidative damage and lipid peroxidation. Additionally, DDT’s ability to reduce iron accumulation and lipid peroxidation suggests that it may directly target the oxidative stress pathway. Our findings demonstrated a reduction in MDA levels and an increase in the GSH/GSSG ratio, indicating that DDT enhances antioxidant defenses in neurons. Thus, the dual regulation of ERS and oxidative stress by DDT likely underpins its strong inhibitory effects on ferroptosis.

While our study provides strong evidence for the neuroprotective effects of DDT, several limitations should be considered. First, a detailed toxicological and pharmacokinetic analysis of DDT has not been conducted, which is essential for evaluating its safety, bioavailability, and optimal dosage for clinical application. Second, while our molecular docking experiments indicate specific binding interactions between DDT components and key ferroptosis-regulating proteins, further validation through *in vivo* studies and crystallography is needed to confirm the precise binding modes involved in these interactions. Additionally, future studies should aim to isolate and characterize the active ingredients of DDT to better understand its molecular targets and therapeutic potential.

This study highlights the therapeutic potential of DDT in treating secondary brain injury caused by ferroptosis following ICH and provides new evidence showing that DDT directly mitigates ferroptosis by regulating the PERK/eIF2α/ATF4/CHOP/GPX4 pathway. The ability of DDT to suppress ERS and its downstream effects is particularly noteworthy, given the growing recognition of ERS as a crucial mediator in ferroptosis. These findings contribute to the growing body of literature on ferroptosis in central nervous system diseases and suggest a new avenue for the clinical management of ICH.

## 5 Conclusion

In conclusion, our study provides evidence that ferroptosis plays a crucial role in ICH pathogenesis due to iron accumulation around the hematoma. DDT effectively mitigates iron deposition and lipid peroxidation, thereby suppressing ferroptosis through regulation of the PERK/eIF2α/ATF4/CHOP/GPX4 signaling pathway. Moreover, the therapeutic effects of DDT in treating ICH are attributed to the synergistic action of key bioactive DDT components, such as sennoside B, amygdalin, rhein, and emodin, which target core proteins in this pathway. Overall, our findings highlight the therapeutic potential and underlying mechanisms of DDT in alleviating ICH-induced neuronal damage, which may also have implications for other central nervous system disorders involving ferroptosis.

## Data Availability

The raw data supporting the conclusions of this article will be made available by the authors, without undue reservation.

## Supplementary material

SUPPLEMENTARY FIGURE 1 Determination of DDT drug concentration. **(A)** The cytotoxic effects of DDT in PC12 cells with different concentrations (0.78, 1.56, 3.125, 6.25, 12.5, 25, 50,100, and 200 μg/mL) was determined by a CCK8 assay. **(B, C)** Hemin- and erastin-induced Fe^2+^ accumulation in PC12 cells was evaluated using the FeRhoNox-1 fluorescent probe, followed by Flow CytoMetry. The bar chart shows the mean values of intracellular Fe2+ accumulation.

## Abbreviations

DDT, Didang Tang; ICH, Intracerebral hemorrhage; HPLC, High performance liquid chromatography; FCM, Flow cytometry; HE, Hematoxylin, and eosin; GSH-px, Glutathione peroxidase; GSH, Glutathione; GSSG, Oxidized glutathione; MDA, Malondialdehyde; SOD, Superoxide dismutase; HO-1, Heme oxygenase; ERs, Endoplasmic reticulum stress; GPX4, Glutathione peroxidase 4; SLC7A11, Solute carrier family 7a member 11; GRP78, Glucose-regulated protein 78; CHOP, C/EBP homologous protein; ATF4, Activating transcription factor-4; eIF2α, Eukaryotic initiation factor 2 alpha; PERK, Protein kinase RNA-like ER kinase.
