# Supplementary figures and images for: Didang Tang alleviates neuronal ferroptosis after intracerebral hemorrhage by modulating the PERK/eIF2α/ATF4/CHOP/GPX4 signaling pathway
Source: Front Pharmacol. 2024 Oct 24;15:1472813. doi: 10.3389/fphar.2024.1472813 (PMC11544539; doi:10.3389/fphar.2024.1472813)

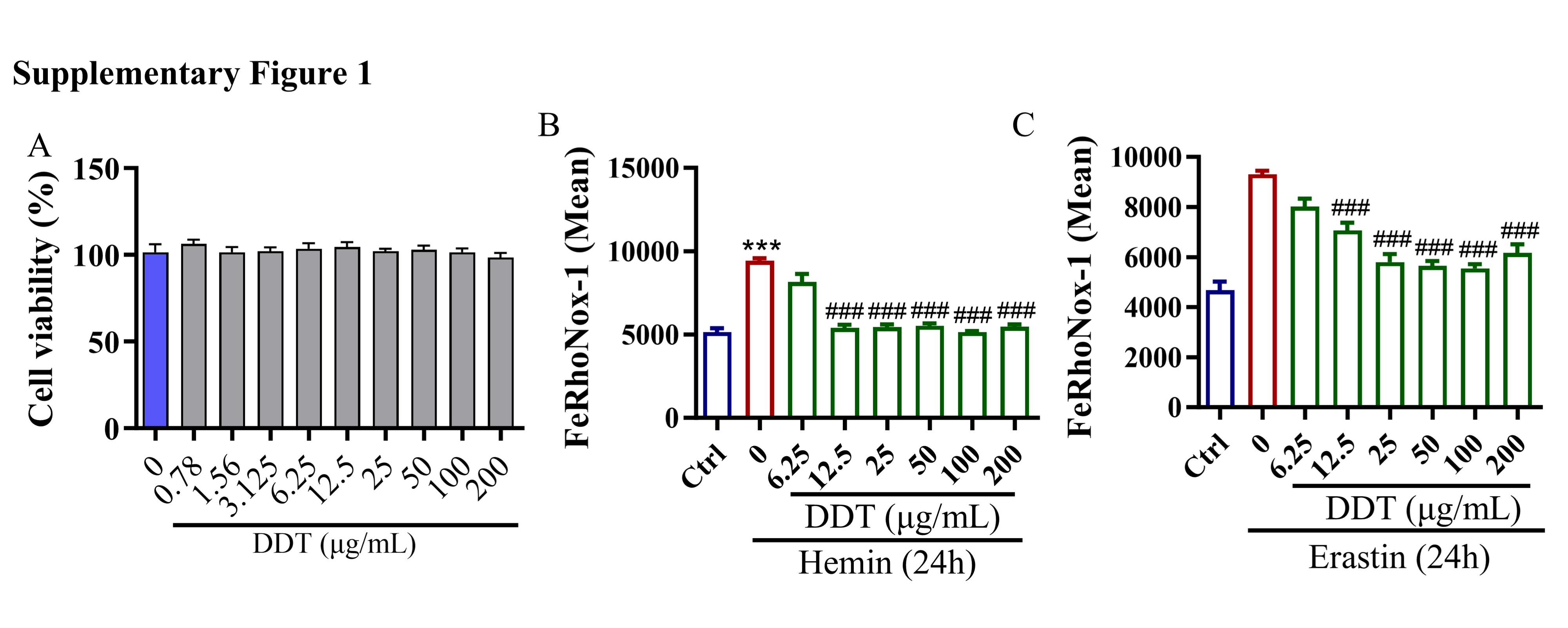

Supplement: Supplementary file 1 [file Image1.TIF]
